# Clinicopathological findings of pediatric *NTRK* fusion mesenchymal tumors

**DOI:** 10.1186/s13000-020-01031-w

**Published:** 2020-09-21

**Authors:** Jeongwan Kang, Jin Woo Park, Jae-Kyung Won, Jeong Mo Bae, Jaemoon Koh, Jeemin Yim, Hongseok Yun, Seung-Ki Kim, Jung Yoon Choi, Hyoung Jin Kang, Woo Sun Kim, Joo Heon Shin, Sung-Hye Park

**Affiliations:** 1 Department of Pathology, Seoul National University Children’s Hospital, College of Medicine, 103 Daehak-ro, Jongno-gu, Seoul, 110-799 Republic of Korea; 2 Precision Medicine, Seoul National University Children’s Hospital, College of Medicine, Seoul, South Korea; 3 Neurosurgery, Seoul National University Children’s Hospital, College of Medicine, Seoul, South Korea; 4 Pediatrics, Seoul National University Children’s Hospital, College of Medicine, Seoul, South Korea; 5 Radiology, Seoul National University Children’s Hospital, College of Medicine, Seoul, South Korea; 6 Lieber Institute for Brain Development, Johns Hopkins Medical Campus, Baltimore, MD 21205 USA; 7 Department of Neurology, Johns Hopkins School of Medicine, Baltimore, MD 21205 USA; 8 Neuroscience Institute, Seoul National University College of Medicine, Seoul, South Korea

**Keywords:** Infantile fibrosarcoma, Undifferentiated sarcoma, *TPR-NTRK1*, *LMNA-NTRK1*, *ETV6-NTRK3*

## Abstract

**Background:**

While *ETV6- NTRK3* fusion is common in infantile fibrosarcoma, *NTRK1/3* fusion in pediatric tumors is scarce and, consequently, not well known. Herein, we evaluated for the presence of *NTRK1/3* fusion in pediatric mesenchymal tumors, clinicopathologically and immunophenotypically.

**Methods:**

We reviewed nine *NTRK* fusion-positive pediatric sarcomas confirmed by fluorescence in situ hybridization and/or next-generation sequencing from Seoul National University Hospital between 2002 and 2020.

**Results:**

One case of *TPR*-*NTRK1* fusion-positive intracranial, extra-axial, high-grade undifferentiated sarcoma (12-year-old boy), one case of *LMNA-NTRK1* fusion-positive low-grade infantile fibrosarcoma of the forehead (3-year-old boy), one case of *ETV6-NTRK3* fusion-positive inflammatory myofibroblastic tumor (IMT) (3-months-old girl), and six cases of *ETV6-NTRK3* fusion-positive infantile fibrosarcoma (median age: 2.6 months, range: 1.6–5.6 months, M: F = 5:1) were reviewed. The Trk immunopositivity patterns were distinct, depending on what fusion genes were present. We observed nuclear positivity in *TPR-NTRK1* fusion-positive sarcoma, nuclear membrane positivity *in LMNA-NTRK1* fusion-positive sarcoma, and both cytoplasmic and nuclear positivity *in ETV6-NTRK3* fusion-positive IMT and infantile fibrosarcomas. Also, the *TPR-NTRK1* fusion-positive sarcoma showed robust positivity for CD34/nestin, and also showed high mitotic rate. The *LMNA-NTRK1* fusion-positive sarcoma revealed CD34/S100 protein/nestin/CD10 coexpression, and a low mitotic rate. The IMT with *ETV6-NTRK3* fusion expressed SMA. Six infantile fibrosarcomas with *ETV6-NTRK3* fusion showed variable coexpression of nestin (6/6)/CD10 (4/5)/ S100 protein (3/6).

**Conclusions:**

All cases of *NTRK1* and *NTRK3* fusion-positive pediatric tumors robustly expressed the Trk protein. A Trk immunopositive pattern and CD34/S100/nestin/CD10/SMA immunohistochemical expression may suggest the presence of *NTRK* fusion partner genes. *LMNA-NTRK1* fusion sarcoma might be a low-grade subtype of infantile fibrosarcoma. Interestingly, more than half of the infantile fibrosarcoma cases were positive for S100 protein and CD10. The follow-up period of *TPR-NTRK1* and *LMNA-NTRK1* fusion-positive tumors are not enough to predict prognosis. However, *ETV6-NTRK3* fusion-positive infantile fibrosarcomas showed an excellent prognosis with no evidence of disease for an average of 11.7 years, after gross total resection of the tumor.

## Background

Next-generation sequencing (NGS) studies have recently revealed an increasing number of fusion genes in soft tissue sarcomas; these genes have been identified as oncogenic drivers and diagnostic markers of a wide range of adult and pediatric cancers [1]. However, until now, the clinicopathological characteristics of all these gene fusion tumors have not been clarified.

Among these recent discoveries are neurotrophic receptor kinase (*NTRK*) gene fusions. *NTRK1*, *NTRK2*, and *NTRK3* encode the neurotrophic tyrosine kinase receptor family TrkA, TrkB, and TrkC transmembrane proteins [2]. These genes play an essential role in nervous system development and function through activation by neurotrophins [3]. However, *NTRK* gene fusions transcribe to chimeric Trk proteins either by constitutive activation or overexpression of kinase-conferring oncogenic proteins [2]. The erythroblast transformation-specific (ETS) variant 6 (*ETV6)-*neurotrophic receptor kinase *(NTRK3)* fusion has been identified in glioblastoma, mammary secretory carcinoma, salivary gland mammary carcinoma, adult lung cancer, papillary thyroid cancer, and mesenchymal tumors including infantile fibrosarcoma, mesoblastic nephroma, IMT, and gastrointestinal stromal tumors [1, 2, 4–8]. Echinoderm Microtubule Associated Protein like-4 (*EML4)-NTRK3* fusion has also been identified in infantile fibrosarcomas and congenital mesoblastic nephroma, in addition to *ETV6-NTRK3* fusion [9]. The common fusion partners of *NTRK1*, located on 1q25, are the 5′ exons of various thyroid-expressed genes (tropomyosin 3 [*TPM3*], translocated promoter region, nuclear basket protein [*TPR*], and *TRK-*fused gene [*TFG*]) in the frame on 1q21–23 because *NTRK1* is located close to its gene partners [10]. Additional fusion partners of *NTRK1* include RAB GTPase activating protein 1-like (*RABGAP1L)*, chromatin target of Protein Arginine Methyltransferase 1 *(PRMT1)* (*CHTOP*), Rho-Rac guanine nucleotide exchange factor 2 (*ARHGEF2*), neurofascin (*NFASC*), and brevican (*BCAN*) [11].

*TPR*-*NTRK1* fusion has been identified in infantile fibrosarcoma [8], pediatric papillary thyroid carcinomas [12], lipofibromatosis [13, 14], interdigitating dendritic cell sarcoma [5], fibrosarcoma-like uterine undifferentiated sarcomas [1], and colorectal adenocarcinomas (Supplementary file: Table 2) [15]. However, it has never been reported in primary intracranial tumors [5]. Additionally, *lamin A/C (LMNA)-NTRK1* fusion has been infrequently reported in congenital infantile fibrosarcoma [4, 16–18], cellular mesoblastic nephroma [19], and lipofibromatosis-like neural tumors [14].

We have recently encountered pediatric cases of intracranial and forehead sarcomas. Pathologically, they did not fit into any known category of sarcomas or benign mesenchymal tumors. However, RNA sequencing by NGS of our cases revealed the presence of *TPR-NTRK1*, *LMNA-NTRK1,* and *ETV6-NTRK3* fusions. Herein, we report these notable cases in detail so that their clinicopathological characteristics can be defined.

## Materials and methods

### Patients

Nine pediatric *NTRK* fusion-positive sarcomas were retrieved from the archives of the Department of Pathology, Seoul National University Children’s Hospital from 2002 to 2019. The fusion genes were detected by either fluorescence in situ hybridization (FISH) or NGS, such as RNA sequencing or customized gene panel study. One case of *ETV6-NTRK3* fusion-positive IMT, one case of *TPR-NTRK1* fusion-, one case of LMNA-NTRK1 fusion- and six cases of *ETV6-NTRK3* fusion-positive sarcomas were reviewed.

### Pathology, immunohistochemistry (IHC), and FISH

All tumors were reviewed by two pathologists (JWK and SHP). IHC stains were performed on an immunostaining system (BenchMark ULTRA system, Ventana-Roche, Mannheim, Germany) using primary antibodies including Trk (1: 50, Cell signaling, Boston, USA), nestin (1: 200, Millipore, Temecula, USA), vimentin (1: 500, DAKO, Grostrup, Denmark), S100 protein (1: 3000, DAKO), CD34 (1: 200, Dako), CD10 (RTU, Novocastra, Newcastle, UK), Ki67 (1: 100, MAb MIB-1; Dako), phosphohistone-H3 (pHH3, 1: 5000, Cell Marque, Rocklin, USA), Transducin-like enhancer of split 1 (TLE1, 1: 20, Cell Marque, Rocklin, US), Fli1 (1: 300, Becton and Dickinson, Flanklin Lakes, US), p53 (1: 100, DAKO), ERG (rtu, Ventana, Export, US), CD99 (1: 200, Novocastra (Leica), Muchen, Germany), smooth muscle actin (SMA, 1: 500, DAKO), desmin (1:200, DAKO), myogenin (1: 500, DAKO), cytokeratin (CK, 1: 300, DAKO), epithelial membrane antigen (EMA, 1: 300, DAKO), integrase interactor 1 (INI-1, 1: 100, Cell signaling,), and Signal transducer and activator of transcription 6 (STAT6, 1: 100, ABCAM, Cambridge, UK) (Suppelementary Table 1). Appropriate positive controls were included, and for the negative control, primary antibodies were omitted. Mitotic activity was assessed with pHH3 immunostain on 4 μm thick formalin-fixed, paraffin-embedded (FFPE) slides by counting mitotic figures in 10 high power fields (HPF; area, 2.38 mm^2^).

For *ETV6* break-apart FISH study, locus-specific identifier (LSI) Vysis *ETV6* fluorescence dual-color break apart DNA probes, *ETV6* [Centromeric (CEN)] SpectrumGreen and Vysis LSI *ETV6* [Telomeric (TEL)] SpectrumOrange (Abbott Molecular, Abbott Park, US) were used.

### DNA extraction and customized brain tumor gene panel study

On hematoxylin and eosin-stained FFPE sections, representative areas of tumors with at least 90% tumor cell purity were outlined for microdissection. DNA-extraction from the serial sections of the microdissected tumor tissue using the Maxwell® RSC DNA FFPE Kit (Promega, USA) was carried out according to the manufacturer’s instructions.

The customized targeted gene panel (FIRST brain tumor panel and FIRST pan-cancer panel), which was customized and verified by the Department of Pathology of Seoul National University Hospital (SNUH), was used, containing 172 genes and ten fusion genes, and with a 1.7 Mb/run by NextSeq550Dx in Hi-Output. The produced sequencing data was analyzed using the pipeline of SNUH First Brain Tumor Panel Analysis. First, we performed the quality control of the Fastq file and analyzed only the data that passed the criteria. Paired-end alignment to the hg19 reference genome was performed using BWA-men and the GATK Best Practice [20]. After finishing the alignment step, an “analysis-ready BAM” was produced, and second quality control was performed to determine if further variant calling is appropriate. In the pipeline, single nucleotide variation (SNV), insertion and deletion (InDel), copy number variation (CNV), and translocation, were analyzed using at least more than two analysis tools, including in-house and open-source software. The open-source tools used were GATK UnifiedGenotyper, SNVer and LoFreq for SNV/InDel detection [21], Delly and Manta for Translocation discovery [22], THetA2 for purity estimation, and CNVKit for CNV calling [23], respectively. SnpEff was used to annotate the variants detected from various databases such as RefSeq, COSMIC, dbSNP, ClinVar, and gnomAD. The germline variant was then filtered using the population frequency of these databases (> 1% population frequency). Finally, the variants were confirmed through a comprehensive review of a multidisciplinary molecular tumor board.

### RNA extraction, RNA sequencing, and fusion analysis

For RNA sequencing, the tumor RNA was extracted from the paraffin block (tumor fraction: > 90%) with Maxwell® RSC RNA FFPE Kit (Promega, USA). The library was generated with SureSelectXT RNA Direct Kit (Agilent, Santa Clara, USA), and sequenced on an Illumina NovaSeq 6000 at Macrogen (Seoul, Republic of Korea). Raw sequencing reads were analyzed with three kinds of algorithms, namely: DIFFUSE, Fusion catcher, and Arriba (https://github.com/suhrig/arriba/), to detect gene fusions. The results were then compared.

Fastq files were briefly aligned by the STAR aligner on the hg19 reference genome for Arriba analysis. The chimeric alignments file and the read-through alignments file were produced, and fusion candidates were generated with a set of filters that detect artifacts based on various characteristic features.

## Result

### Clinicopathological findings and follow-up data of the patients

**Fig. 1:**
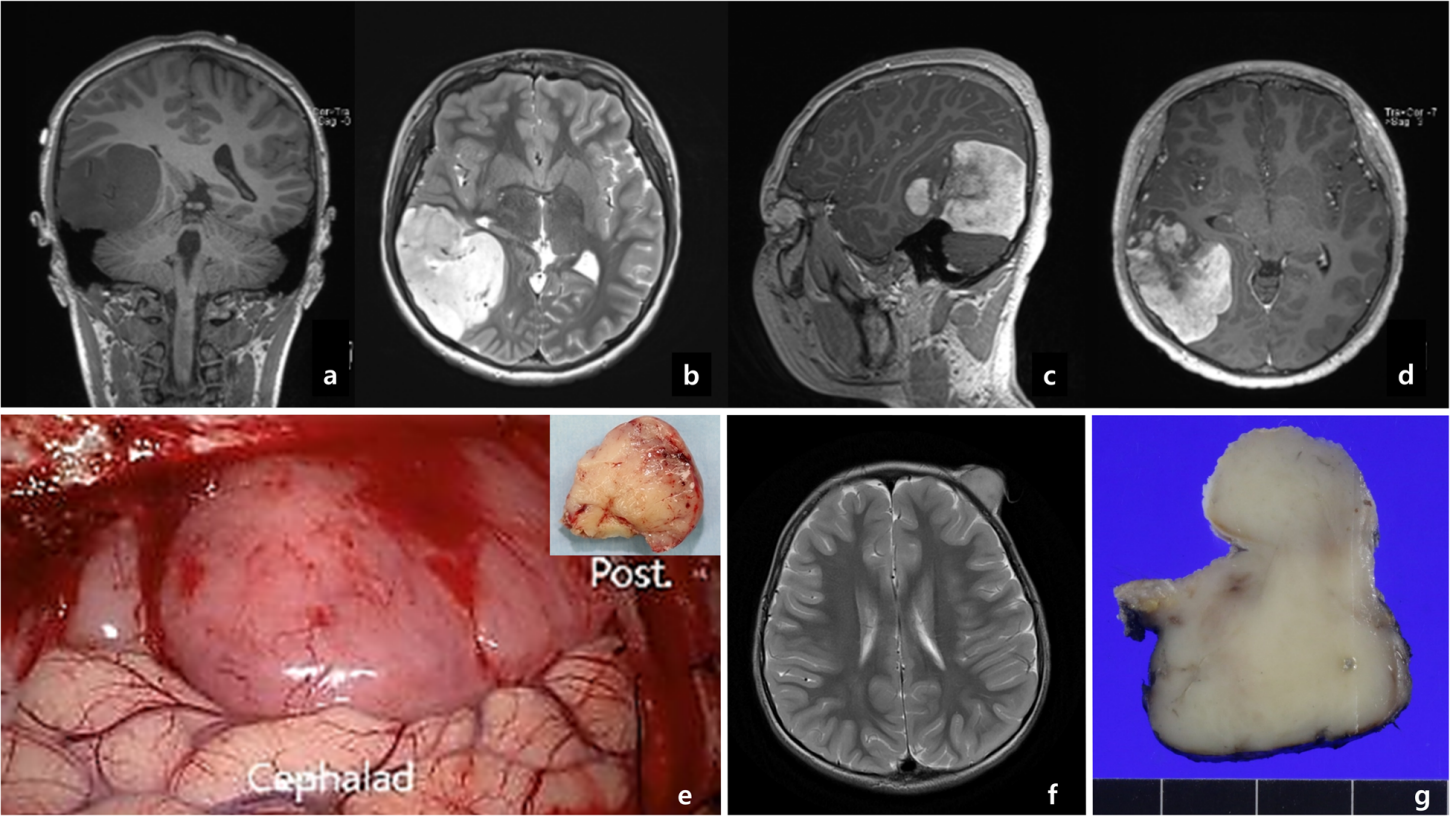
**a**-**e** Case 1 with *TPR*-*NTRK1* fusion.: MRI reveals **a**) T1-low, **b-d**) T2-high dura-based mass with enhancement. **e** The tumor was located in the right temporal convexity and right cerebellar tent (The direction of the brain mentioned as cephalhead and posterior). The inlet is the cut surface of the tumor showing yellowish, and solid without hemorrhage or necrosis. **f**, **g** Case 2 with *LMNA-NTRK1* fusion tumor: T2 weighted MRI revealed low-density mass on the left forehead. The cut surface of the tumor shows a gray-white solid appearance without hemorrhage or necrosis

The patient with *TPR-NTRK1* fusion-positive sarcoma was a 12-year-old boy who presented with headache and diplopia for 3 months, and did not have any perinatal health problems. A 7.4-cm contrast-enhancing mass was detected in the right temporal lobe on magnetic resonance imaging (MRI) (Fig. 1a-d). Craniotomy revealed a hypervascular, extra-axial tumor with superficial brain invasion (Fig. 1e). Complete resection of the tumor with adjuvant chemotherapy with Ifosfamide, Carboplatin, and Etoposide (*ICE*) and radiation therapy (54 + 7.2 Gy) were administered because the pathology was high-grade undifferentiated sarcoma.

The patient with *LMNA-NTRK1* fusion-positive sarcoma was a 3-year-old boy who presented with a growing mass on his left forehead, which had been present since he was a neonate as a pea-sized mass, and it has recently grown rapidly to 4.0 × 3.5 × 3.0 cm. It protruded from the forehead and was covered with eroded skin. The patient underwent complete surgical excision, and the cut surface of the tumor exhibited a homogenous tan-colored solid appearance (Fig. 1f-g).

**Fig. 2:**
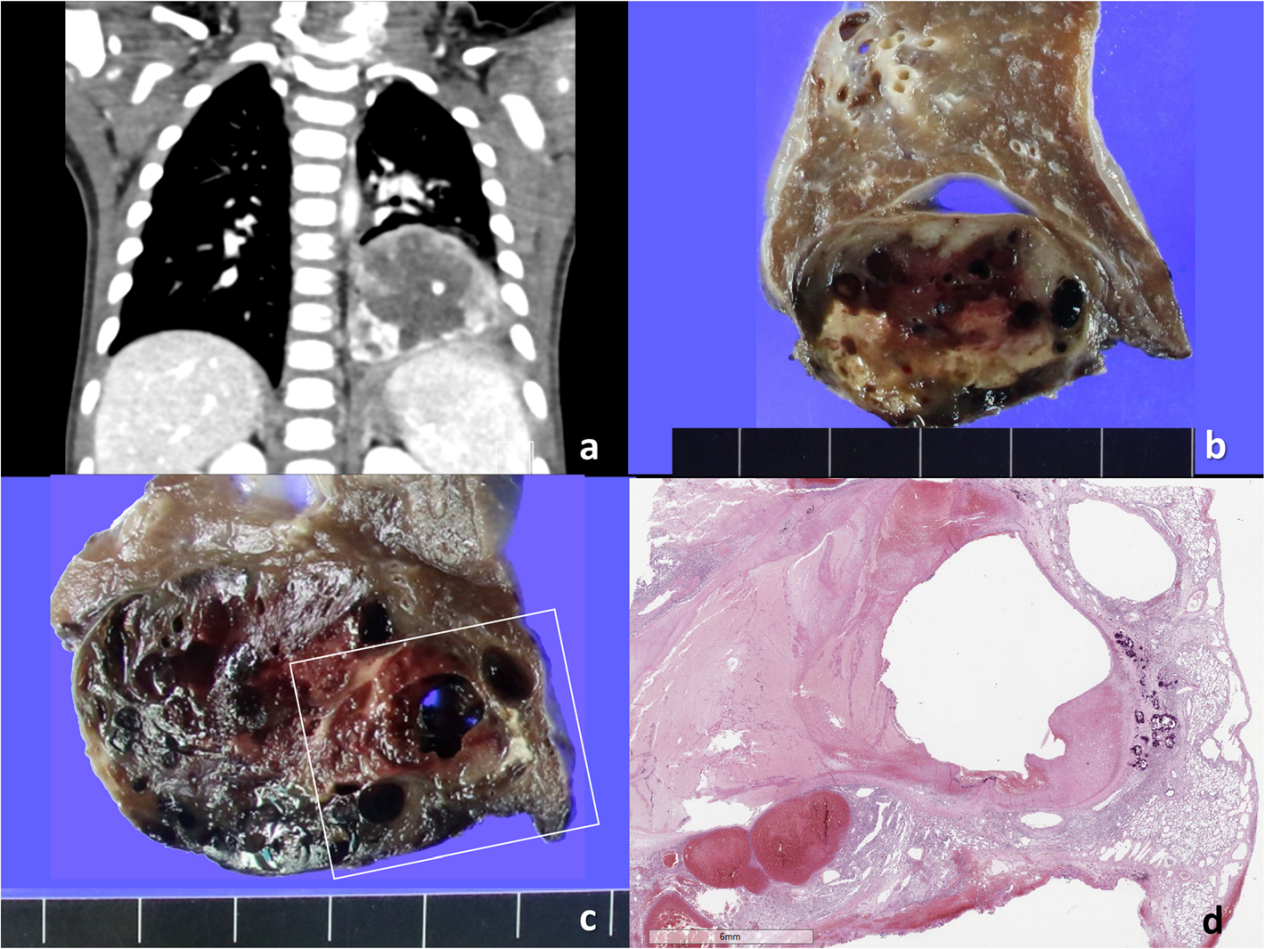
**a** Chest CT of the *ETV6-NTRK3* fusion-positive inflammatory myofibroblastic tumor (IMT) reveals a heterogeneously enhancing tumor in the left lower part of the thorax. **b** The mass arises from extrapulmonary sequestration, supplied by the left phrenic artery, which is separated from the lower lobe of the lung. **c** The cut surface of the tumor is hemorrhagic and has congested large vessels. **d** This is the microscopic picture of the squared part of Fig. **c**. It is a well-encapsulated, but partly adhered to the lower lobe of the left lung. The tumor pushed the left lower lobe of the lung. Hemorrhage was developed by previous embolization of large vessels of the sequestrated lung, under the impression of arteriovenous malformation

The patient with *ETV6-NTRK3* fusion-positive IMT was a 3-month-old girl who presented with sudden onset dyspnea and systemic cyanosis. Chest computerized tomography (CT) showed a mass on the left lower thorax, that looked like a mass of the lower lobe of the left lung (Fig. 2). The mass was embolized under the impression of arteriovenous malformation at the local hospital. However, the symptom and signs were not relieved, and the mass had grown continuously to 5.6 × 5.2 × 3.3 cm. Lobectomy of the left lower lobe was then conducted to remove the tumor. Grossly, the mass was well-encapsulated and well-separated from the left lower lobe of the lung (Fig. 2). The tumor arose from an extrapulmonary sequestration, and was diagnosed as IMT by full pathological examination and NGS (using the customized First pan-cancer gene panel).

The median age of the six *ETV6-NTRK3* fusion-positive infantile fibrosarcoma patients were 2.6 months (range: 1.6–5.6 months of age) at the time of surgery. The male to female ratio was 5: 1. The patients had presented with a mass on the tongue, buttock, right shoulder, left foot, right abdominal cavity, and sacrococcygeal area, respectively. Five tumors were completely resected, and adjuvant chemotherapies were given, as summarized in Table 1. The remaining massive sacrococcygeal tumor, involving the spinal cord, was initially subtotally resected and underwent three operations with one cycle of chemotherapy, but the patient was lost to follow-up.

**Table 1:** Clinicopathological comparison of our two cases of *TPR-NTRK1* and *LAMA-NTRK1* fusion-positive sarcomas and 6 cases of our infantile fibrosarcoma

	*TPR-NTRK1* fusion-positive pediatric sarcoma	*LMNA-NTRK1* fusion-positive pediatric sarcoma	*ETV6-NTRK3* fusion-positive Inflammatory myofibroblastic tumor	*ETV6-NTRK3* fusion-positive pediatric sarcoma
Age/Gender	12 y/ male	3 y/ male	6 mo/female	Median age: 2.6 months (range: 1.6 ~ 5.6 months), M: F = 5:1
Site	Dura, parieto-occipital	Forehead dermis and subcutaneous tissue	Left lower lobe of lung	Tongue, buttock, shoulder, foot, abdominal cavity, and sacro-coccygeal area
Size	6.0 × 5.0 × 3.0 cm	4.0 × 3.5 × 3.0 cm	5.6 × 3.5 × 3.0 cm	Range: 1.4–4.5 cm in biggest diameter
Histology	Mixed oval to spindle cells	Spindle cells intermixed with prominent lymphoplasma cells	Spindle myofibroblastic cells with prominent lymphoplasma cells	Mostly spindle cells, with no prominent inflammatory cell infiltration
Histological grade	High-grade	Low-grade	Low-grade	High-grade (cellular)
Nuclear pleomorphism	Relatively uniform cells without pleomorphism	Uniform cells without pleomorphism	Uniform cells without pleomorphism	Uniform cells without pleomorphism
Tumor necrosis	Absent	Absent	Abent	Absent
Mitotic rate	25/10 HPFs	0/10 HPFs	1/ 10 HPFs	10/10HPFs ~ 40/10 HPFs
Ki67 labeling index	36.0%	18.2%	37%	15 ~ 60%
Immuno-positive markers	Trk/CD34/Nestin/vimentin/p53	Trk/ S100/CD34/Nestin/vimentin/	Trk/SMA/vimentin	Trk/Nestin/vimentin, S100 (3/6 cases)/CD10(4/5 cases)
Trk positive pattern	Nucleus	Nuclear membrane	Cytoplasmic	Cytoplasm and nucleus
Genetic abnormalities other than NTRK1 fusion	NTRK1 amplification (copy number: 11), H3F3A amplification (copy number: 12)	absent	absent	Absent
Final diagnosis	Undifferentiated sarcoma	Infantile fibrosarcoma, low grade	Inflammatory myofibroblastic tumor	Infantile fibrosarcoma, cellular
Treatment	Surgery+ vincristine, doxorubicin, cyclophosphamide (6 cycles)/ifosramide, carboplatin, etoposide (6 cycles), alternative, total 12 cycles	Surgery only	Surgery only	Surgery + vincristine, actinomycin D, cyclophosphamide (X11 or 26 cycles)
Outcomes and follow-up	NED (1.4 years)	NED (0.8 years)	NED (0.3 years)	5 patients: NED (for average 11.7 years, range: 6.0–17.4 years) 1 patient: Follow-up loss
Known mesenchymal tumors with this type of fusion	Lipofibromatosis, adult uterine undifferentiated sarcoma	Infantile fibrosarcoma, lipofibromatosis-like neural tumor, Undifferentiated sarcoma, Cellular mesoblastic nephroma	Infantile fibrosarcoma	Infantile fibrosarcoma, inflammatory myofibroblastic sarcoma

*NED* No evidence of disease

The follow-up data are summarized in Table 1. The patients with *TPR-NTRK1* and *LMNA-NTRK1* fusion-positive sarcomas fared relatively well, with no tumor recurrence or neurological defects, during the 18 months and 11.6 months follow-up period, respectively. Five patients with *ETV6-NTRK3* fusion-positive infantile sarcomas are all alive without disease for an average of 11.7 years (range: 6.0–17.4 years), but one case who had a huge sacrococcygeal mass was lost to follow-up.

### Result of pathology, IHC, and FISH

**Fig. 3:**
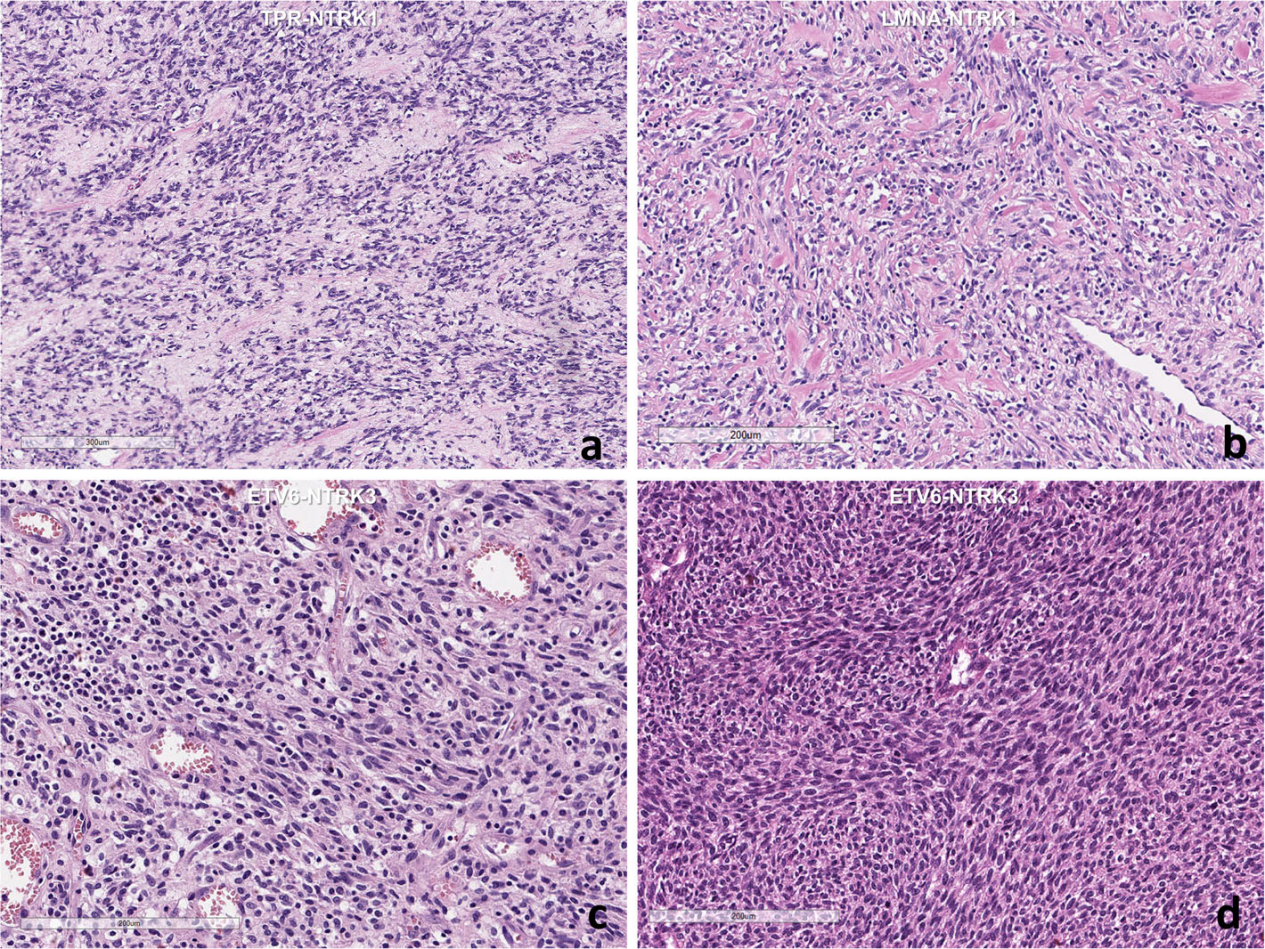
**a** Histology of the intracranial undifferentiated sarcoma with *TPR-NTRK1* fusion shows alternating cellular areas with collagen bands show a tiger pattern-like appearance. The tumor cells are oval to short spindle cells. **b** The forehead mesenchymal tumor with *LMNA-NTRK1* fusion shows relatively low cellular spindle cell mesenchymal tumor with keloid type collagen laydown. **c** The pulmonary inflammatory myofibroblastic tumor with *ETV6-NTRK3* fusion shows bland-looking spindle cells with intermixed lymphoplasma cells. **d** A sacrococcygeal infantile fibrosarcoma with *ETV6-NTRK3* fusion shows fascicular spindle cells with high cellularity (**a-d**: H&E, bar: **a**: 300, μm, **b-d**: 50 μm)

**Fig. 4:**
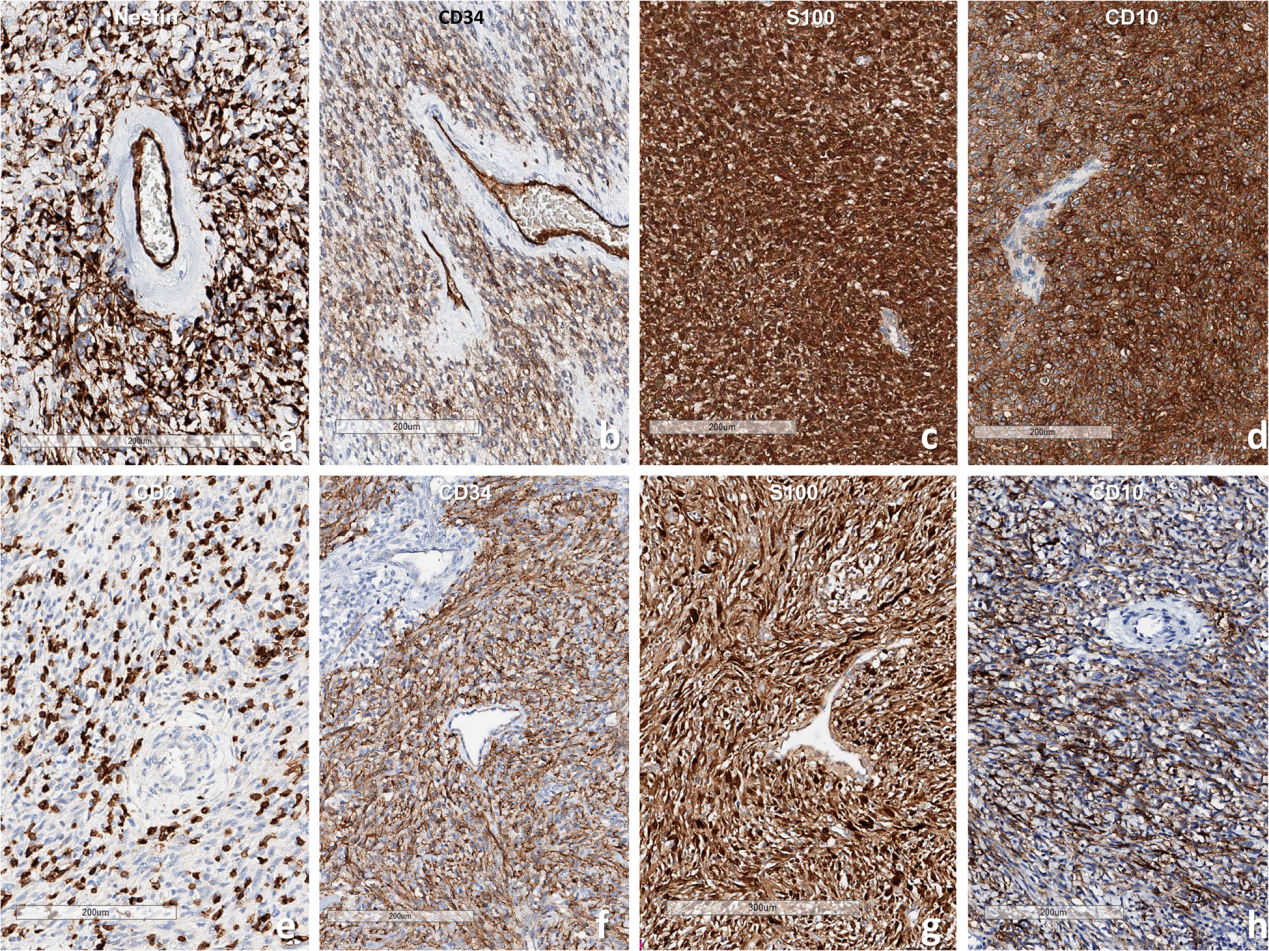
**a**, **b** Sarcoma with *TPR-NTRK1* fusion shows co-positive for nestin and CD34. **c**, **d** *ETV6-NTRK3* fusion-positive infantile fibrosarcoma is co-positive for S100 protein and CD10. **e**-**h** The *LMNA-NTRK1* fusion sarcoma has lots of CD3-positive T-cell infiltration and robustly coexpressed CD34, S100, and CD10. (**a**: nestin, **b, f**: CD34, **c, g**: S100 protein, **e**: CD34D, **h**: CD10, Bar: 200 μm)

**Fig. 5:**
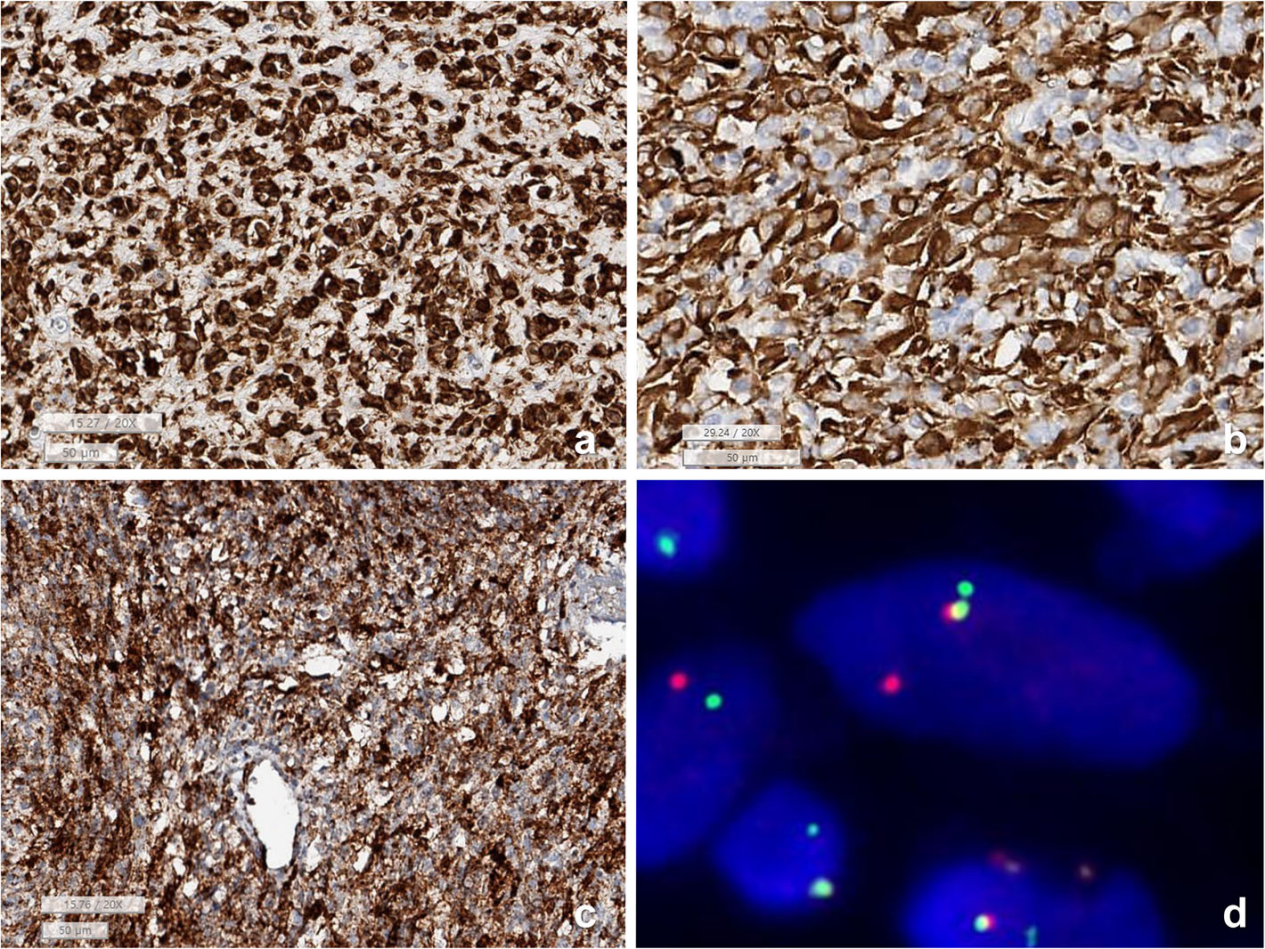
Trk immunohistochemistry shows **a** Nuclear positivity in *TPR-NTRK1* fusion sarcoma (Case 1), **b** mostly nuclear membrane, and cytoplasmic stain in *LMNA-NTRK1* fusion sarcoma (Case 2). and **c** mainly cytoplasmic stain in *ETV6-NTRK3* fusion sarcoma. In Fig. **b**, the Trk-negative cells are infiltrating inflammatory cells (**a-c**: Trk IHC, lower bar: 50 μm). **d**. locus-specific identifier (LSI) FISH study using *ETV6* fluorescence dual-color break apart DNA probes show one fused and one widely separated SpectrumGreen and SpectrumOrange signals in an infantile fibrosarcoma with *ETV6-NTRK3* fusion-positive

Histopathology of the *TPR-NTRK1* fusion-positive sarcoma showed a sheet of small oval-to-spindle cells with dilated blood vessels. Scanning power microscopy revealed a tiger-striped pattern due to vague layers of cellular and less-cellular areas with keloid type collagen deposits (Fig. 3). The tumor cells exhibited relatively uniform oval nuclei with fine chromatin and clear-to-eosinophilic cytoplasm. A high mitotic rate (25/10 per high-power fields) and a high Ki-67 labeling index (36.0%) were present; however, necrosis was not observed. The tumor cells were also robustly positive for Trk (1: 50, Cell Signaling, Boston, US), CD34, nestin, p53, and vimentin (Fig. 4). The robust nuclear positivity of Trk was remarkable (Fig. 5). However, the tumor cells were negative for S-100 protein, SMA, desmin, myogenin, CD99, Fli-1, CD56, STAT6, CK and EMA. TLE1 was weakly positive for the tumor cell nuclei and INI1 was retained.

The *LMNA-NTRK1* fusion-positive tumor was composed of vaguely fascicular spindle cells with bland-looking elongated nuclei and inconspicuous nucleoli (Fig. 3c). There was collagen laid down between the tumor cells. Intermixed inflammatory cell infiltration was remarkable, which was pronounced on CD3 IHC (Fig. 4e). The Ki-67 index was moderately high (18.2%), but many of them might be infiltrating inflammatory cells. Mitosis was absent on pHH3 IHC. There was neither necrosis nor hemorrhage. Therefore, this tumor was much less cellular and much more bland-looking than *TPR-NTRK1* or *ETV6-NTRK3* fusion-positive sarcoma. The tumor cells were robustly and diffusely positive for Trk, S100-protein, CD34, and nestin (Figs. 4 and 5), but negative for CD56, SMA, desmin, myogenin, STAT6, EMA, CK, CD1a, CD21, CD35, CD43, WT-1(c-terminal), MelanA, HMB45, BRAF, and ALK. The nuclear envelope-positivity for Trk was remarkable with weak cytoplasmic staining (Fig. 3).

The *ETV6-NTRK3* fusion-positive IMT was composed of vaguely fascicular bland-looking spindle cells intermixed with lymphoplasma cells (Figs. 2 and 3). The tumor cells were positive for Trk and SMA, but negative for S100, CD34, ALK, CD10, desmin, myogenin, CD99, CD56, CK, EMA, and STAT6. There was no necrosis. Mitosis was very low (1/10HPF), but the Ki-67 index was 36%, possibly due to the presence of inflammatory cells. *ETV6* break FISH was positive and pan-cancer panel resulted in *ETV6-NTRK3* fusion (split read: 339, spaning read: 40).

The histopathology of *ETV6-NTRK3* fusion-positive sarcomas showed highly cellular and relatively uniform small spindle cells with a high mitotic rate (10–40/10 HPFs). There was neither necrosis nor prominent inflammatory cell infiltration in all cases. These infantile fibrosarcomas were diffusely and robustly positive for Trk (100%), S100 protein (50%, 3/6 cases), nestin, CD10 (80%, 4/5 cases), and vimentin (100%), but negative for CD34, SMA, desmin, myogenin, and CD56. The Trk IHC showed a diffuse cytoplasmic stain with some nuclear staining (Fig. 5). Ki-67 labeling indices were 15–60%. *ETV6-NTRK3* fusion was verified by FISH in all six cases (Fig. 5) and crosschecked by RNA sequencing in two cases.

**Fig. 6:**
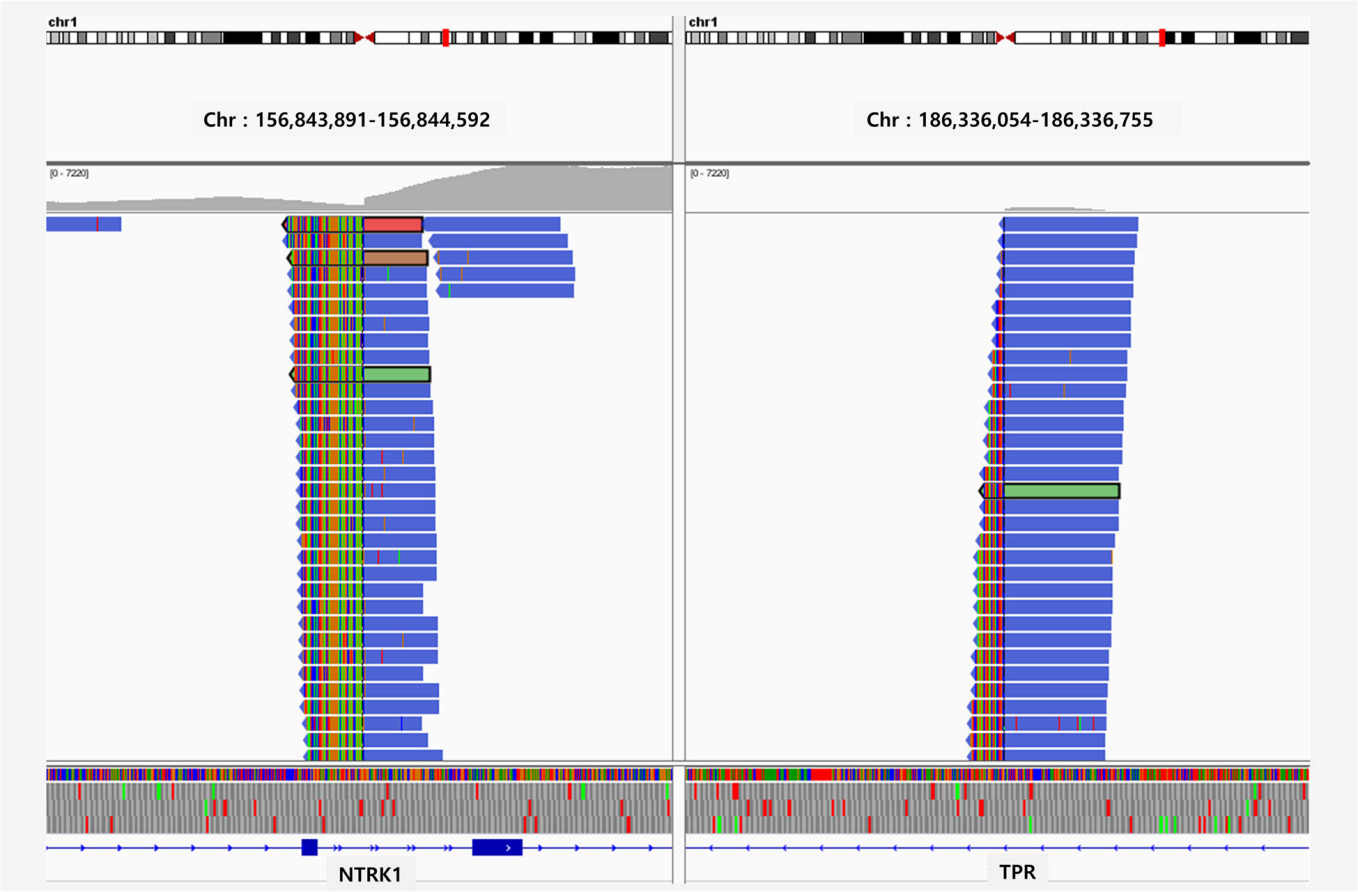
The custom NGS panel revealed *TPR-NTRK1* fusion (IGV capture)

*TPR-NTRK1* fusion was double-checked by targeted DNA gene panel and RNA sequencing. The targeted gene panel revealed a *TPR*-*NTRK1* fusion of *TPR* on chromosome 1q25 (position 186,337,018) and *NTRK1* on chromosome 1q21-q22 (position 156,844,363) with amplification of *NTRK1* (copy number: 11) and *H3F3A* (copy number: 12) on chromosome 1 in case 1 (Fig. 6).

RNA sequencing of an intracranial sarcoma (12-year-old boy) confirmed the presence of *TPR-NTRK1* fusion (Breakpoint: 1: 186337018, 1: 156844363). RNA sequencing of a forehead tumor (3-year-old boy) confirmed the presence of *LMNA-NTRK1* fusion (Breakpoint: 1: 156104766, 1: 156844698). The number of split reads in *TPR* and *NTRK1* was 35 and 31, respectively, with two discordant mates, and 37 split reads in *LMNA* and 53 in *NTRK1*, with seven discordant mates. RNA sequencing performed in two cases of infantile fibrosarcoma showed *ETV6-NTRK3* fusion (Case 4). The breakpoints and split reads of *ETV6* and *NTRK3* (Breakpoints: 12: 12022903: 15: 88483984, 12: 12022903, 15: 88524591) were 11 and 16, and 25 and 8, respectively (Supplementary Fig. 1-4). Split reads are the read fragments of the unmatched paired-end alignments. A *discordant* alignment happens when both *mates* align uniquely, but does not satisfy the paired-end constraints.

## Discussion

Primitive small round cell sarcomas and infantile fibrosarcomas are rare childhood sarcomas that pose diagnostic and therapeutic challenges. Recently, confirmative diagnosis of neoplasms has been made possible at the genomic level by identification of driver mutation or marker gene alterations [24]. Recent reports have described emerging pediatric fusion-positive sarcomas, including *NTRK* [5, 8, 25, 26]. Our *NTRK* fusion-positive pediatric sarcomas have distinct immunohistochemical profiles. The *TPR-NTRK1* fusion-positive tumor was a CD34-positive, dural-based, high-grade undifferentiated sarcoma with features that did not fit the classifications of existing types of sarcoma. In contrast, our *LMNA-NTRK1* fusion-positive tumor was a low-grade spindle cell mesenchymal tumor of the forehead that was first noticed early in the neonatal period. The *LMNA*-*NTRK1* fusion-positive tumor was difficult to diagnose before RNA sequencing by NGS because of its unusual pathology and immunohistochemical profile, namely, a combination of prominent inflammatory cells, no mitotic activity (0/10 HPF), and S100/CD34 coexpression. However, Hung et al.’s case of infantile fibrosarcoma also showed prominent inflammatory cells [4]. S100-protein and CD34 co-positivity are generally rare in sarcomas; these can be interpreted as hybridomas or evidence of dual differentiation. However, infantile fibrosarcomas often show coexpression of these two antibodies [16, 27]. Miettinen et al. and Wong et al. reported a non-pleomorphic, low-grade spindle cell neoplasm with *LMNA-NTRK1* fusion, that was diagnosed as infantile fibrosarcoma [17, 27]. Miettinen et al. ‘s case showed low mitotic rates (< 5/10 HPFs), and S100 protein/CD34-coexpression [27]. Wong et al.’s case was CD34/vimentin-positive [17]. Our *LMNA-NTRK1* fusion-positive sarcoma was consistent with Hung et al.’s and Miettinen et al.’s infantile fibrosarcoma with S100 protein/CD34 coexpression. The main differential diagnosis of this *LMNA-NTRK1* fusion tumor was IMT because of prominent inflammatory cells in the tumor, but it can be ruled out based on its immunoprofile (SMA-negative, with S100/CD34 coexpression).

*NTRK1* encodes *TRKA* receptor tyrosine kinase, which has a high affinity for nerve growth factor [3]. Genetic alterations of *NTRK1* by translocations, amplifications, deletions, and point mutations have been reported in various tumor types, suggesting the potential role of Trk in oncogenesis [28, 29]. More recently, *NTRK1* chromosomal rearrangements have been identified in additional tumor types (Supplementary file, Table 2) [10, 21, 30, 31], suggesting that while oncogenic activation through *NTRK1* fusion is not frequent, it can occur in various cancers. Interestingly, a significant number of *NTRK1*-associated gene fusions have developed as a result of intrachromosomal gene fusion [11]. Depending on the directions of transcription of *NTRK1* and its fusion partner, intrachromosomal fusions can occur either through simple interstitial deletion (e.g., *LMNA-NTRK1*) or through a more complex break/inversion mechanism (e.g., *TPM3-NTRK1* or *TPR-NTRK1*), if the two genes are transcribed in opposite directions [13]. A 737-kbp deletion yielded the 5′ end of *LMNA* (localized to 1q22), including exons 1–10 fused to the 3′ end of *NTRK1* (also localized to 1q22) and exons 12–17 [17].

Pan-Trk IHC can be used to detect *NTRK* fusion tumors; however, the expression site within the tumor cell differs according to the fusion partner genes [4]. We found strong nuclear envelope and cytoplasmic positivity in our *LMNA-NTRK1* fusion-positive tumor. Intense nuclear staining in our *TPR-NTRK1* fusion-positive sarcoma was observed with Trk (clone A7H6R) IHC, which is consistent with Hechtman et al. ‘s report using monoclonal antibody [MAb] EPR17341 [32]. However, a diffuse and strong cytoplasmic staining with MAb EPR17341 was reported in both *LMNA-NTRK1* fusion-positive tumor and *TPM3-NTRK1* fusion-positive sarcoma [1, 33]. Davis et al. reported nuclear positivity in *NTRK3* fusion tumors and cytoplasmic positivity in *NTRK1/2* fusion tumors using the panTrk IHC (EPR17341) [8]. These differences in immunopositivity might be due to different Trk antibody clones and different types of sarcomas.

*ETV6-NTRK3* and (rarely) *EML4-NTRK3, LMNA-NTRK1, TPM3-NTRK1,* and *SQSTM1-NTRK1* fusions have been reported in infantile fibrosarcomas (Table 1) [4, 8, 16]. The six cases of classic infantile fibrosarcoma and one IMT in our study had an *ETV6-NTRK3* fusion verified by *ETV6* break-apart FISH and/or RNA sequencing. The diffuse cytoplasmic Trk positivity in our cases is consistent with the Trk immunopositivity patterns in *ETV6*-*NTRK3* fusion-positive tumors from previous reports [4, 27].

Although *ETV6-NTRK3* is a genetic hallmark of infantile fibrosarcoma, it has also been reported in ALK-negative IMTs. So far, six cases of *ETV6-NTRK3* fusion-positive IMTs have been published [5–7]. Chang et al. reported that ALK-altered thoracic IMTs were 73% (24/33), and the remaining ALK-negative IMTs had *ROS1* fusion (15%, 5 cases) or *ETV6-NTRK3* fusion (9%, 3 cases) or *RET* fusion (3%, 1 case) [6]. Our IMT case is unique because it occurred in the extrapulmonary sequestered lung, had *ETV6-NTRK3* fusion, and is the youngest reported *ETV6-NTRK3* fusion-positive IMT in the literature [34]. The previously reported youngest patient with *ETV6-NTRK3* fusion-positive IMT was 2 years old [6].

These *NTRK* fusion tumors tend to respond to *NTRK* inhibitors [2, 11]. LOXO-101 is an orally bioavailable tyrosine kinase inhibitor that inhibits Trk catalytic activity with a low nanomolar potency. A phase 1 study with LOXO-101 in soft tissue sarcoma with *LMNA–NTRK1* fusion and non-small cell lung cancer harboring *TPR-NTRK1* fusion showed a good response [35, 36]. Crizotinib was a durable response in the *LMNA-NTRK1* fusion-positive undifferentiated pleomorphic sarcoma [37]. *NTRK* gene fusion could be a novel target of *NTRK* inhibitors for multiple tumor types [2].

In conclusion, we report two cases of *NTRK1* fusion-positive and seven cases of *NTRK3* fusion-positive pediatric sarcomas and IMT that were diagnostically challenging without molecular features. These three types of fusion-positive mesenchymal tumors (*TPR-NTRK1*, *LMNA-NTRK1,* and *ETV6-NTRK3*) differed in their H&E morphology, immunoprofile, and Trk immunopositivity patterns. In the case of *LMNA-NTRK1* fusion sarcoma, S100/CD34/CD10-coexpression was a novel finding. The S100 protein, nestin, and CD10 positivity in infantile fibrosarcoma was also a new finding. The *TPR-NTRK1* fusion sarcoma was positive for CD34 and nestin but negative for S100 protein. Thus, the Trk and CD34/S100/nestin/CD10 immunophenotype could be used for differential diagnosis. The sacrococcygeal infantile fibrosarcoma was unable to achieve complete resection, and the exact outcome is unknown because the patient was lost to follow-up. However, the remaining patients with *ETV6-NTRK3* fusion-positive infantile fibrosarcomas survived for up to 17.3 years (median survival: 8.3 years), without tumor recurrence, after complete resection of the tumor. The patients with these fusion-positive tumors may benefit from *NTRK* inhibitor therapy if the tumors cannot be controlled by conventional treatment [38].

## Supplementary information

**Additional file 1: Supplementary Table 1**. Primary antibodies using this study. **Supplementary Table 2**. Summary of previously reported mesenchymal tumors with NTRK1 fusion.

**Additional file 2: Supplementary Figure 1A.** RNA sequencing confirmed *TPR-NTRK1* fusion using the Arriba fusion gene calling method. 1) Circular plot. 2) The fusion gene retained the protein tyrosine kinase domain. 3) The schematic view showed *TPR-NTRK1* fusion by 488 bp deletion (breakpoints: chromosome 1: 186337018; 1: 156844363). **Supplementary Figure 1B.** RNA sequencing confirmed *LMNA-NTRK1* fusion using the Arriba fusion gene calling method (breakpoints: chromosome 1: 156104766; 1: 156844698). 1) The schematic view showed *LMNA-NTRK1* fusion. 2) Circular plot. 3) The fusion gene retained the protein tyrosine kinase domain. **Supplementary Figure 1C.** RNA sequencing confirms *ETV6-NTRK3* fusion in the fifth case of infantile fibrosarcoma using the Arriba fusion gene calling method. 1) The schematic view showed *ETV6-NTRK3* fusion (breakpoints: chromosome 12: 12022903; 15: 88483984). 2) Circular plot. 3) The fusion gene retained the protein tyrosine kinase domain. **Supplementary Figure 1D.** RNA sequencing confirmed *ETV6-NTRK3* fusion in the 6th case of infantile fibrosarcoma using the Arriba fusion gene calling method. 1) The schematic view showed *ETV6-NTRK3* fusion (breakpoints: chromosome 12: 12022903; 15: 88524591). 2) Circular plot. 3) The fusion gene retained the protein tyrosine kinase domain.

## Data Availability

All the genetic data can be found in our SNUH’s big data server managing by the center for precision medicine.

## Abbreviations

ARHGEF2: Rho-Rac guanine nucleotide exchange factor 2
BCAN: Brevican
CEN: Centromeric
CHOP: Chromatin target of PRMT1
CNV: Copy number variation
CT: Computeriaed tomography
ETV6: Ets variant 6
EML4: Echinoderm Microtubule Associated Protein like-4
FFPE: Formalin-fixed paraffin-embedded
FISH: Fluorescence in situ hybridization
HPF: High-power field
*ICE*: Ifosfamide, carboplatin, and etoposide
IHC: Immunohistochemical
IMT: Inflammatory myofibroblastic tumor
InDel: Insertion and deletion
LMNA: Lamin A/C
LSI: Locus-specific identifier
Mab: Monoclonal antibody
MRI: Magnetic resonance imaging
NFASC: Neurofascin
NGS: Next-generation sequencing
NTRK: Neurotrophic receptor kinase
PRMT1: Protein Arginine Methyltransferase 1
RABGAP1L: RAB GTPase activating protein 1-like
SNUH: Seoul National University Hospital
SNV: Single nucleotide variation
TEL: Telomeric
TFG: TRK-fused gene
TPM3: Tropomyosin 3
TPR: Translocated promoter region, nuclear basket protein
