# Supplementary material for: Clinicopathological findings of pediatric NTRK fusion mesenchymal tumors
Source: Diagn Pathol. 2020 Sep 21;15:114. doi: 10.1186/s13000-020-01031-w (PMC7507612; doi:10.1186/s13000-020-01031-w)
Supplement: Supplementary file 1 — Additional file 1: Supplementary Table 1. Primary antibodies using this study. Supplementary Table 2. Summary of previously reported mesenchymal tumors with NTRK1 fusion. [file 13000_2020_1031_MOESM1_ESM.docx]

Supplementary Table 1. Primary antibodies using this study.

| Antibody | Dilution | Source |
| --- | --- | --- |
| TRK | 1: 50 | Cell signaling, Boston, USA |
| Nestin | 1: 200 | Milipore, Temecula, USA |
| Vimentin | 1: 500 | DAKO, Glostrup, Denmark |
| S-100 protein | 1: 3000 | DAKO, Glostrup, Denmark |
| CD34 | 1: 200 | DAKO, Glostrup, Denmark |
| CD10 | rtu | Novocastra (Leica), Muchen, Germany |
| Ki-67 | 1: 100 | DAKO, Glostrup, Denmark |
| pHH3 | 1: 100 | Cell Marque, Rocklin, USA |
| TLE1 | 1: 50 | Cell marque, , Rocklin, USA |
| FLI1 | 1: 300 | Becton and Dickinson, Flanklin Lakes, USA |
| p53 | 1: 100 | DAKO, Glostrup, Denmark |
| ERG | rtu | Ventana, Export, US |
| CD99 | 1: 200 | Novocastra (Leica), Muchen, Germany |
| Smooth muscle actin | 1: 500 | DAKO, Glostrup, Denmark |
| Desmin | 1: 200 | DAKO, Glostrup, Denmark |
| Myogenin | 1: 500 | DAKO, Glostrup, Denmark |
| Olig2 | 1: 500 | Cell Marque, Rocklin, USA |
| GFAP | 1: 200 | DAKO, Glostrup, Denmark |
| CD56 | 1: 100 | Cell Marque, Rocklin, USA |
| CD68 | 1 :2000 | DAKO, Glostrup, Denmark |
| CD21 | 1 :50 | Novocastra (Leica), Muchen, Germany |
| CD35 | 1: 100 | Novocastra (Leica), Muchen, Germany |
| Cytokeratin | 1 ; 300 | DAKO, Glostrup, Denmark |
| Epithelial membrane antigen | 1: 300 | DAKO, Glostrup, Denmark |
| Integrase interactor 1 (INI-1) | 1: 100 | Cell Marque, Rocklin, US |
| Lin-28 homolog A (Lin28A) | 1:100 | Cell signaling, Danver, US |
| STAT6 | 1: 100 | ABCAM, Cambridge, UK |

+: positive, -: negative, rtu: ready to use, ND: not done

Supplementary Table 2. Summary of previously reported mesenchymal tumors with NTRK1 fusion.

|  | NTRK fusion | Age/gender | Diagnosis | Pathology comments | S100/CD34 IHC | Reference |
| --- | --- | --- | --- | --- | --- | --- |
| 1 | TPR-NTRK1 | 12y/M | Intracranial (dural) undifferentiated sarcoma | Small round to short spindle cell undifferentiated sarcoma | S100+/CD34f+ | Kang et. al. 2019 (Present case) |
| 2 | TPR-NTRK1 | 7y/F, 14y/F | Lipofibromatosis | Locally aggressive lipofibromatosis-like Neural Tumor | S100+/CD34 sparse+ | Agaram et al. 2016 (13), Bartenstein et al. 2018 (14) |
| 3 | TPR intron21-NTRK1 intron 9 | 20y/M | Soft tissue histiocytic or dendritic cell neoplasm | Interdigitating dendritic cell sarcoma | NM | Pavlick et al. 2017 (5) |
| 4 | TPR-NTRK1 | 27y, 42y, 46, 47y/F | Uterine sarcoma | Uterine undifferentiated sarcoma with fibrosarcoma-like morphology | S100+/CD34- | Chiang et al. 2018 (1) |
| 5 | LMNA-NTRK1 | 1mo/ | Congenital/infantile fibrosarcoma | Low grade spindle cell sarcoma without atypia or mitotic activity | S100 (NM)/CD34+ | Wong et al. 2015 (17) |
| 6 | LMNA-NTRK1 | 2mo/F | Infantile fibrosarcoma | Low grade spindle cell sarcoma without atypia or mitotic activity | S100+/CD34+ | Davis et al. 2018 (16) |
| 7 | LMNA-NTRK1 | 14mo/M | Lipofibromatosis-like neural tumor | Fascicular spindled tumor without atypia or mitotic activity | S100+/ CD34 sparse + | Bartenstein et al. 2018 (14) |
| 8 | LMNA-NTRK1 | 13d/F | Metastatic infantile fibrosarcoma | Low-grade fibromatosis-like tumor with fascicular myxoid tumor without atypia or mitotic activity | S100+/SMA+ | Bender et al. 2019 (18) |
| 9 | LMNA-NTRK1 | 1y/M | Soft tissue sarcoma | Metastatic fibrosarcoma | NM | Pavlick et al. 2017 (5) |
| 10 | LMNA-NTRK1 | 41Y/F | Soft tissue sarcoma | Undifferentiated sarcoma | NM | Doebele et al. 2015 (34) |
| 1 | LMNA-NTRK1 | 45y/M | Lumbosacral pleomorphic undifferentiated sarcoma | High-grade spindle cell sarcoma with MDM2 amplification | S100 f+/CD34- | Zhou et al. 2018 (35) |
| 12 | LMNA-NTRK1 | 1mo/M | Infantile fibrosarcoma | Low grade spindle cell sarcoma without atypia or mitotic activity | S100+/CD34+ | Kang et al. (present case) |
| 13 | SQSTM1-NTRK1 | 0/F | Fibrosarcoma | NM | NM | Pavlick et al. 2017 (5) |
| 14 | SQSTM1-NTRK1 | <5y/F | Fibrosarcoma | NM | NM | Doebele et al. 2015 (34) |
| 15 | TPM3-NTRK1 | 2mo/M | Infantile fibrosarcoma | NM | NM | Davis et al. 2019 (8) |
| 16 | TPM3-NTRK1 | 0/M | LG spindle cell sarcoma | NM | NM | Davis et al. 2019 (8) |
| 17 | TPM3-NTRK1 | 0/M | LG spindle cell sarcoma/HG at recurrence | NM | NM | Davis et al. 2019 (8) |
| 18 | TPM3-NTRK1 | 0/M | Unclassified | NM | NM | Davis et al. 2019 (8) |
| 19 | TPM3-NTRK1 | 18mo/M | Infantile fibrosarcoma | NM | NM | Davis et al. 2019 (8) |
| 20 | TPM3-NTRK1 | 36mo/F | LG spindle cell tumor | NM | NM | Davis et al. 2019 (8) |
| 21 | TPM3-NTRK1 | 120M/M | Inflammatory spindle and round cell sarcoma | NM | NM | Davis et al. 2019 (8) |
| 22 | TPM3-NTRK1 | 2mo/M | Inflammatory fibroid polyp | NM | NM | Davis et al. 2019 (8) |
| 23 | TPM3-NTRK1 | 0/M | Spindle cell sarcoma | NM | NM | Davis et al. 2019 (8) |

IHC: immunohistochemistry, NM: not mentioned
