# Supplementary material for: Clinicopathological findings of pediatric NTRK fusion mesenchymal tumors
Source: Diagn Pathol. 2020 Sep 21;15:114. doi: 10.1186/s13000-020-01031-w (PMC7507612; doi:10.1186/s13000-020-01031-w)
Supplement: Supplementary file 2 — Additional file 2: Supplementary Figure 1A. RNA sequencing confirmed TPR-NTRK1 fusion using the Arriba fusion gene calling method. 1) Circular plot. 2) The fusion gene retained the protein tyrosine kinase domain. 3) The schematic view showed TPR-NTRK1 fusion by 488 bp deletion (breakpoints: chromosome 1: 186337018; 1: 156844363). Supplementary Figure 1B. RNA sequencing confirmed LMNA-NTRK1 fusion using the Arriba fusion gene calling method (breakpoints: chromosome 1: 156104766; 1: 156844698). 1) The schematic view showed LMNA-NTRK1 fusion. 2) Circular plot. 3) The fusion gene retained the protein tyrosine kinase domain. Supplementary Figure 1C. RNA sequencing confirms ETV6-NTRK3 fusion in the fifth case of infantile fibrosarcoma using the Arriba fusion gene calling method. 1) The schematic view showed ETV6-NTRK3 fusion (breakpoints: chromosome 12: 12022903; 15: 88483984). 2) Circular plot. 3) The fusion gene retained the protein tyrosine kinase domain. Supplementary Figure 1D. RNA sequencing confirmed ETV6-NTRK3 fusion in the 6th case of infantile fibrosarcoma using the Arriba fusion gene calling method. 1) The schematic view showed ETV6-NTRK3 fusion (breakpoints: chromosome 12: 12022903; 15: 88524591). 2) Circular plot. 3) The fusion gene retained the protein tyrosine kinase domain. [file 13000_2020_1031_MOESM2_ESM.pdf]

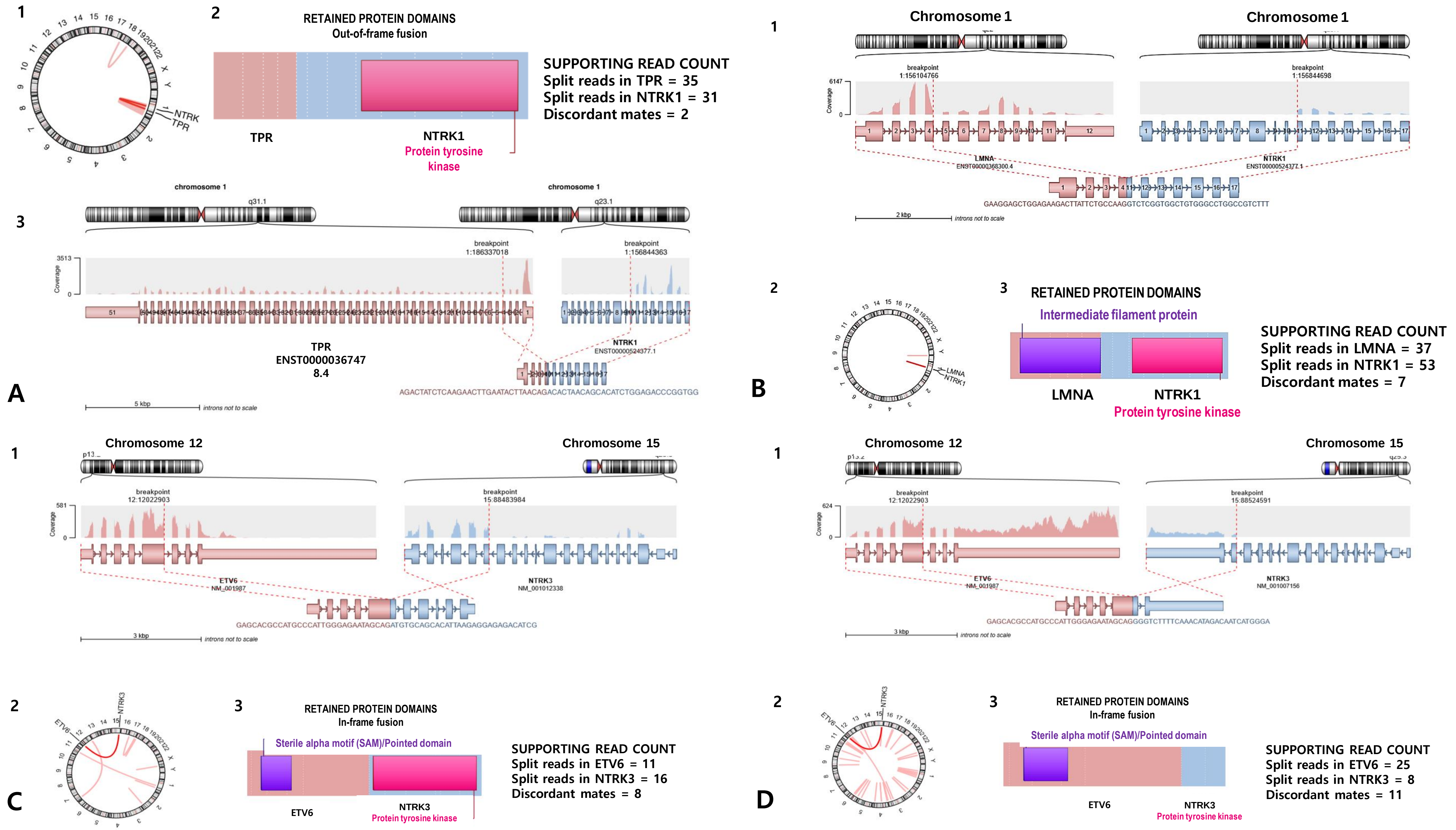
A
1
NTRK
TPR
2
RETAINED PROTEIN DOMAINS
Out-of-frame fusion
TPR
NTRK1
Protein tyrosine kinase
SUPPORTING READ COUNT
Split reads in TPR = 35
Split reads in NTRK1 = 31
Discordant mates = 2
3
chromosome 1
q31.1
chromosome 1
q23.1
breakpoint
1:186337018
breakpoint
1:156844363
3513
Coverage
0
TPR
ENST0000036747
8.4
NTRK1
ENST00000524377.1
AGACTATCTCAAGAACTTGAATACTTAACAGACACTAACAGCACATCTGGAGACCCGGTGG
5 kbp
introns not to scale
B
1
Chromosome 1
Chromosome 1
breakpoint
1:156104766
breakpoint
1:156844698
6147
Coverage
0
LMNA
ENST00000368300.4
NTRK1
ENST00000524377.1
GAAGGAGCTGGAGAAGACTTATTCTGCCAAGGTCTCGGTGGCTGTGGGCCTGGCCGTCTTT
2 kbp
introns not to scale
2
LMNA
NTRK1
3
RETAINED PROTEIN DOMAINS
Intermediate filament protein
LMNA
NTRK1
Protein tyrosine kinase
SUPPORTING READ COUNT
Split reads in LMNA = 37
Split reads in NTRK1 = 53
Discordant mates = 7
C
1
Chromosome 12
Chromosome 15
breakpoint
12:12022903
breakpoint
15:88483984
581
Coverage
0
ETV6
NM_001987
NTRK3
NM_001012338
GAGCACGCCATGCCCATTGGGAGAATAGCAGATGTGCAGCACATTAAGAGGAGAGACATCG
3 kbp
introns not to scale
2
ETV6
NTRK3
3
RETAINED PROTEIN DOMAINS
In-frame fusion
Sterile alpha motif (SAM)/Pointed domain
ETV6
NTRK3
Protein tyrosine kinase
SUPPORTING READ COUNT
Split reads in ETV6 = 11
Split reads in NTRK3 = 16
Discordant mates = 8
D
1
Chromosome 12
Chromosome 15
breakpoint
12:12022903
breakpoint
15:88524591
624
Coverage
0
ETV6
NM_001987
NTRK3
NM_001007156
GAGCACGCCATGCCCATTGGGAGAATAGCAGGGGTCTTTTCAAACATAGACAATCATGGGA
3 kbp
introns not to scale
2
ETV6
NTRK3
3
RETAINED PROTEIN DOMAINS
In-frame fusion
Sterile alpha motif (SAM)/Pointed domain
ETV6
NTRK3
Protein tyrosine kinase
SUPPORTING READ COUNT
Split reads in ETV6 = 25
Split reads in NTRK3 = 8
Discordant mates = 11
